# Reducing health inequality in Black, Asian and other minority ethnic pregnant women: impact of first trimester combined screening for placental dysfunction on perinatal mortality

**DOI:** 10.1111/1471-0528.17109

**Published:** 2022-02-27

**Authors:** Becky Liu, Usaama Nadeem, Alexander Frick, Morakinyo Alakaloko, Amar Bhide, Basky Thilaganathan

**Affiliations:** ^1^ Fetal Medicine Unit St George's University Hospitals NHS Foundation Trust London UK; ^2^ Vascular Biology Research Centre, Molecular and Clinical Sciences Research Institute St George's University of London London UK; ^3^ Tommy's National Centre for Maternity Improvement Royal College of Obstetrics and Gynaecology London UK

**Keywords:** early screening, ethnicity, neonatal death, perinatal death, placental dysfunction, pre‐eclampsia, stillbirth

## Abstract

**Objective:**

To assess the impact of the Fetal Medicine Foundation (FMF) first trimester screening algorithm for pre‐eclampsia on health disparities in perinatal death among minority ethnic groups.

**Design:**

A retrospective cohort study from July 2016 to December 2020.

**Setting:**

A large London teaching hospital.

**Patients and methods:**

All women who underwent first trimester pre‐eclampsia risk assessment using either the NICE screening checklist or the FMF multimodal approach. Women considered at high‐risk in the FMF cohort were offered 150 mg aspirin before 16 weeks' gestation, serial growth scans and elective birth at 40 weeks.

**Main outcome measures:**

Stillbirth, neonatal death and perinatal death rates stratified by screening method and maternal ethnicity.

**Results:**

In the NICE cohort, the perinatal death rate was significantly higher in non‐white than white women (7.95 versus 2.63/1000 births, OR 3.035, 95% CI 1.551–5.941). Following the introduction of FMF screening, the perinatal death rate in non‐white women fell from 7.95 to 3.22/1000 births (OR 0.403, 95% CI 0.206–0.789), such that it was no longer significantly different from the perinatal mortality rate in white women (3.22 versus 2.55/1000 births, OR 1.261, 95% CI 0.641–2.483).

**Conclusions:**

First trimester combined screening for placental dysfunction is associated with a significant reduction in perinatal death in minority ethnic women. Health disparities in perinatal death among ethnic minority women demand urgent attention from both clinicians and health policy makers. The data of this study suggest that this ethnic health inequality may be avoidable.

**Tweetable abstract:**

Multimodal early pregnancy placental dysfunction screening can lead to a significant reduction in perinatal deaths in non‐white women.

## Tweetable abstract

Multimodal early pregnancy placental dysfunction screening can lead to a significant reduction in perinatal deaths in non‐white women.

This article includes Author Insights, a video abstract available at https://vimeo.com/bjogabstracts/authorinsights17109.

## 1 INTRODUCTION

Health inequality for a and other minority ethnic women is an area of public health and intense media interest. Women of Asian and Black origin are known to have higher rates of adverse pregnancy outcomes.^1^, ^2^ A large meta‐analysis demonstrated a two‐fold increased likelihood of stillbirth in black women,^3^ and this health disparity is also reflected in other ethnic and socio‐economically disadvantaged groups.^4^, ^5^, ^6^ Historically, ethnic health disparities were thought to be due to socio‐economic factors and poorer access to healthcare services with possible language barriers.^2^ However, these disparities persist even after taking account of socio‐economic factors and other demographic variables.^7^ Possible reasons include unintentional racial bias, which occurs even though the majority of healthcare professionals neither hold nor endorse such racial stereotypes.^8^ A lack of high‐quality research has led to the proposal of various strategies to tackle ethnic health disparities including induction of labour at term for all ethnic minority women.^9^

To date, these efforts have failed to address the impact of routine early pregnancy risk assessment, which forms the basis for personalising care pathways with the aim of reducing adverse outcomes. However, the recommended National Institute for Health and Care Excellence (NICE) checklist of risk factors does not take maternal ethnicity into consideration, and is known to have a high screen positive rate and a low detection rate.^10^, ^11^ In contrast, the Fetal Medicine Foundation (FMF) first trimester combined screening algorithm for pre‐eclampsia detection has demonstrated superior detection rates with its efficacy established in a randomised controlled trial.^11^, ^12^ This algorithm incorporates maternal ethnicity and produces a numerical risk for the development of placental dysfunction leading to pre‐eclampsia and/or small‐for‐gestational‐age (SGA) birth. The FMF assessment has been effectively incorporated into a routine healthcare setting in our unit, with targeted aspirin prophylaxis, serial third‐trimester ultrasound assessments and earlier term birth offered to at‐risk pregnancies.^13^ This package of care has led to a relative effect reduction of 80% in preterm pre‐eclampsia and 45% in term SGA birth, without increasing healthcare resources.^13^, ^14^

We hypothesise that the observed improved outcomes of pregnancies screened as at high‐risk of placental dysfunction may have translated into improved perinatal outcomes in minority ethnic groups by focusing interventions on the population at higher risk for adverse outcomes based on a validated multi‐marker model. The aim of this study is to assess the impact of the first trimester FMF screening programme for placental dysfunction on perinatal mortality rates across various ethnic groups.

## 2 METHODS

This study analysed all women who received antenatal care at St George's University Hospitals NHS Foundation Trust from July 2016 to December 2020. The FMF screening programme was implemented in April 2018, and consisted of an algorithm‐based risk assessment tool combining maternal history, mean arterial pressure (MAP), pregnancy‐associated plasma protein A (PAPP‐A) and uterine artery pulsatility index (UtA PI). Pregnancies at high‐risk of placental dysfunction were defined by a ≥1:50 chance of preterm pre‐eclampsia. Women in this risk category received prophylactic low dose aspirin (150 mg once daily) until 36 weeks and serial growth ultrasound scans at 28 and 36 weeks, and were offered induction of labour at 40 weeks’ gestation.^13^ Women who were transferred from another care provider in the second or third trimesters were only included if they were transferred more than 4 weeks prior to birth and therefore still received some routine antenatal care in our unit.

### 2.1 Outcome measures

Maternal characteristics included age, height, weight, parity and ethnicity. Ethnicity was self‐certified and considered in four aggregated groups from those used by the Office for National Statistics: White, Black, Asian and Mixed/Other. Fetal/neonatal characteristics for the perinatal deaths were inclusive of estimated fetal weight (EFW), EFW centile and gestational age at birth. Pregnancy complications such as hypertensive disorders of pregnancy, gestational or pre‐existing diabetes, and small‐for‐ gestational‐age birth (defined EFW <10th centile) were also noted for the perinatal deaths.^15^ All perinatal deaths which occurred during the study period were identified—stillbirths were defined as *in utero* demise from 24 weeks' gestation, with a birthweight >300 g. Perinatal deaths were inclusive of stillbirths and neonatal deaths (NND) within 28 days of birth. All cases were verified for accuracy through individual medical records. Women who underwent termination of pregnancy or had a fetus with a major congenital anomaly and/or genetic or chromosomal abnormality where death may not have been preventable, were excluded from the analysis. Women with SGA fetuses received serial growth scans every 2 weeks from 28 weeks, but if fetal growth restriction (FGR) was diagnosed (AC or EFW <10th centile and umbilical artery PI or uterine artery PI >95th centile), surveillance took place according to the TRUFFLE protocol with weekly scans and alternate day computerised cardiotocography.^16^ Delivery was planned according to EFW centile and Doppler findings.^16^, ^17^

### 2.2 Statistical analysis

Descriptive data were presented in median and interquartile range for continuous variables and in numbers and percentages for categorical variables. Comparisons between groups were performed using the Mann–Whitney *U*‐test for continuous variables and the Ch‐square test or Fisher's Exact test for categorical variables. The statistical software package SPSS 27.0 (SPSS Inc.) and R version 4.0.1 (2020‐06‐06) were used for data analyses.

## 3 RESULTS

During the study period, 20 651 women underwent routine first trimester screening: 8080 with conventional NICE criteria and 12 571 with FMF combined screening for placental dysfunction after April 2018. No clinically significant differences were seen in the maternal characteristics for women who were screened using the NICE or FMF algorithms (Table 1) except for a small but statistically significant increase in women classified as mixed/other in the FMF screened cohort.

**TABLE 1:** Table displaying the maternal characteristics of women who underwent routine first trimester screening using the NICE checklist and the FMF screening algorithm

	NICE screened (*n* = 8080)	FMF screened (*n* = 12 571)	*P*‐value
Age (years)	32.6 (29.2–35.9)	32.8 (29.4–35.9)	0.053
Height (cm)	164.0 (160.0–168.0)	164.0 (160.0–169.0)	0.371
Weight (kg)	65.0 (58.0–74.3)	65.1 (58.3–74.7)	0.244
Nulliparity	3732 (46.2%)	5957 (47.4%)	0.092
Ethnicity			
White	5314 (65.8%)	8222 (65.4%)	0.555
Black	941 (11.7%)	1440 (11.5%)	0.661
Asian	1518 (18.8%)	2330 (18.5%)	0.589
Mixed/other	307 (3.8%)	579 (4.6%)	0.006

### 3.1 Stillbirth, neonatal death and perinatal death rates

There were 179 perinatal deaths during the time period of interest, reduced to 102 after exclusion of pregnancy terminations and known major/lethal congenital structural or genetic abnormalities (Figure S1). With exclusion of *in utero* transfers within 4 weeks of birth, 55 stillbirths (2.66/1000 births) and 16 NNDs (0.77/1000 births), in all 71 perinatal deaths (3.44/1000 births), were screened and received antenatal care at St George's Hospital and were included in the final analysis (Table 2). Some of the perinatal deaths were known to be complicated by SGA (*n* = 15), diabetes (*n* = 12) or pre‐eclampsia (*n* = 5). In the NND group, 12 (75.0%) were complicated by preterm birth (<37 weeks), of which 11 (68.8%) were very preterm (<34 weeks).

**TABLE 2:** Table comparing the prevalence of stillbirth, neonatal death and perinatal death and acquired pregnancy complications in women who underwent NICE and FMF screening

Outcomes	NICE screened (*n* = 8080)	FMF screened (*n* = 12 571)	Odds ratio (95% CI)
Perinatal deaths	36 (4.46/1000 births)	35 (2.78/1000 births)	**0.625 (0.391–0.994)**
SGA and/or HDP	14 (38.9%)	6 (17.2%)	**0.275 (0.106–0.716)**
Stillbirths	26 (3.22/1000 births)	29 (2.31/1000 births)	0.716 (0.422–1.217)
SGA and/or HDP	8 (30.7%)	4 (13.7%)	0.321 (0.097–1.067)
Neonatal deaths	10 (1.24/1000 births)	6 (0.48/1000 births)	0.385 (0.140–1.061)
SGA and/or HDP	6 (60.0%)	2 (33.3%)	0.214 (0.043–1.061)
Preterm birth <34 weeks	6 (60.0%)	5 (83.3%)	0.535 (0.163–1.755)

Bold values are statistically significant. Cases with confirmed small‐for‐gestational‐age birth (SGA), hypertensive disorders of pregnancy (HDP) or preterm birth are shown as sub‐groups.

### 3.2 FMF screening and perinatal death rates by ethnicity

**FIGURE 1:**
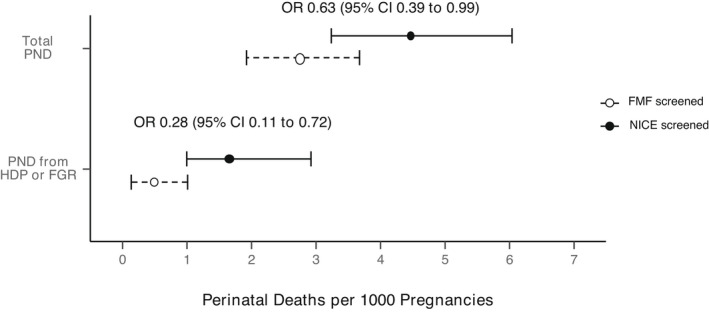
Graph showing absolute rates (95% confidence intervals) of perinatal death in the women who underwent NICE and FMF screening, stratified by whether there was a diagnosis of hypertensive disorders of pregnancy (HDP) and/or fetal growth restriction (FGR)

**FIGURE 2:**
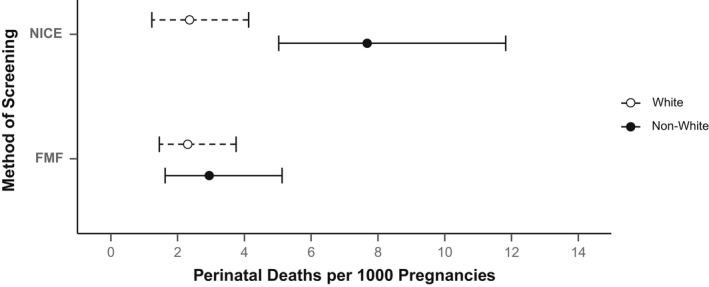
Graph showing absolute rates (95% confidence intervals) of perinatal death in the White and non‐White populations, stratified by method of screening

First trimester FMF combined screening for placental dysfunction was associated with a significant reduction (OR 0.625, 95% CI 0.391–0.994) in perinatal deaths from 4.46/1000 births in the NICE cohort to 2.78/1000 births in the FMF cohort (Table 2, Figure 1). The overall fall in perinatal death rate was also associated with a significant decrease in perinatal deaths associated with SGA and/or hypertensive disorders of pregnancy (HDP) (OR 0.275, 95% CI 0.106–0.716). There were decreases in stillbirth and neonatal death rates between the two cohorts, but this did not reach statistical significance. In the NICE screened cohort, the perinatal death rate was significantly higher in non‐White than White ethnic groups (7.95 versus 2.63/1000 births; OR 3.035, 95% CI 1.551–5.941; Table 3, Figure 2, Figure S2). Following the introduction of the first trimester FMF screening programme for placental dysfunction, the perinatal death rate fell in the non‐White group from 7.95 to 3.22/1000 births (OR 0.403, 95% CI 0.206–0.789) but did not fall in the White group (OR 0.969, 95% CI 0.493–1.908). There were reductions in perinatal death rates observed across all minority ethnic groups, with the Asian group reaching statistical significance (OR 0.324, 95% CI 0.121–0.865; Table S1, Figures S2 and S3). There was no significant health inequity for perinatal mortality between the White and non‐White groups in the FMF screened cohort (2.55 versus 3.22/1000 births, OR 1.261, 95% CI 0.641–2.483).

**TABLE 3:** Table comparing the perinatal death rate in women who underwent NICE and FMF screening, stratified by White and non‐White ethnicity

	NICE screened (*n* = 8080)	FMF screened (*n* = 12 571)	Odds ratio (95% CI)
White (*n* = 13 536)	2.63 (1.5–4.4)	2.55 (1.7–4.0)	0.969 (0.493–1.908)
Non‐white ethnicities (*n* = 7115)	7.95 (5.3–12.1)	3.22 (1.9–5.4)	**0.403 (0.206–0.789)**
Odds ratio (95% CI)	**3.035 (1.551–5.941)**	1.261 (0.641–2.483)	

Bold values are statistically significant. The odds ratios (95% CI) are shown by type of screening method and by ethnicity.

## 4 DISCUSSION

Routine pregnancy risk assessment using the NICE checklist was associated with a three‐fold higher perinatal death rate in non‐White than White women. Implementation of FMF first trimester combined screening for placental dysfunction was associated with a 60% decrease of perinatal deaths in non‐White women—equivalent to 21 babies lives saved in 4349 non‐White pregnancies. The reduction in perinatal death resulted from a 73% reduction in deaths linked to HDP and SGA birth. The findings of this study suggest that first trimester combined screening for placental dysfunction may help confer equitable perinatal outcomes for non‐White and White women.

### 4.1 Implications for clinical practice

One previous study evaluated multiple first trimester biophysical and biochemical markers and suggested that a model combining these variables predicted 55% of stillbirths due to placental dysfunction at a false‐positive rate of 10% as in this study.^18^ However, no published studies have evaluated the impact of FMF screening on perinatal death rates, let alone stratified by maternal ethnic groups. We have previously demonstrated that successful implementation of the first trimester FMF combined screening programme into a routine healthcare setting resulted in significant reduction in the prevalence of pre‐eclampsia as well as term SGA birth.^13^, ^14^ This is consistent with the proven superiority of FMF first trimester placental dysfunction screening algorithms compared with the routine use of the NICE risk assessment checklist in detecting these diseases.^19^

The NICE pregnancy risk assessment checklist is limited by several factors:

- different risk criteria are given equal weighting despite different propensities for placental dysfunction,
- continuous variables such as maternal age are used with arbitrary categorical thresholds,
- risk criteria are used to increase but never to reduce the risk,
- interactions between risk factors are not considered,
- maternal ethnicity is not taken into account when assessing pregnancy risk,
- a numerical estimate of risk is not produced, thereby precluding personalisation of care for women.

The use of the FMF screening algorithm accounts for these limitations and allows for the calculation of a personalised risk for placental dysfunction. It is evident that such a screening programme and targeted interventions not only result in improved pregnancy outcomes but could also directly address a large source of maternal health inequality for black, Asian and other minority ethnic women.

The perinatal mortality in white women trended to be reduced by FMF screening, but did not reach statistical significance. This is most likely due to an already very low perinatal death rate in this group of 2.6/1000 pregnancies. It is also noteworthy that just under half of the perinatal deaths in the entire cohort occurred in pregnancies with complications such as pre‐eclampsia or diabetes, suggesting that increased monitoring and early intervention in these women may be justified.^20^ Regular fetal wellbeing assessments such as fetal growth assessment, fetal Doppler and fetal heart rate (FHR) monitoring with computerised cardiotocography (cCTG) may be beneficial in at‐risk pregnancies. However, these methods of surveillance would only provide a brief window of insight into fetal wellbeing during the period of actual monitoring and are likely to be limited by the frequency of appointments and availability of healthcare professionals. With the rapid evolution of telemedicine, the use of a validated and reliable method of remote ambulatory FHR monitoring may well play an increasing role.^21^, ^22^, ^23^

### 4.2 Public health policy implications

Evident health disparities in ethnic minority women are issues that the public health sector is urged to minimise. Although causation for such health disparity is presumed to be complex and related to socio‐economic and cultural factors, the possibility that health care systems and recommendations may actually worsen such disparity, remains poorly investigated. The issue of whether a woman's ethnicity should be included in pregnancy risk assessment is a source of intense debate. This study demonstrates that when a woman's ethnicity is included in a robust, externally validated risk prediction tool, we may significantly improve perinatal outcomes for women of non‐White ethnicity. This finding suggests that perinatal death is not directly attributable to a woman's ethnicity, but that factors such as maternal age, body mass index and ethnicity are proxy markers for the biology of placental dysfunction.

Placental dysfunction in particular, presents specific unrecognised problems to public health specialists, with pre‐eclampsia, SGA birth and stillbirth all being recognised consequences of this disorder. In the UK, the National Screening Committee considers pre‐eclampsia, SGA birth and stillbirth as three different disease entities, whereas composite screening for these three outcomes is likely to be far more effective, as is evident from the use of the FMF screening test in the current study. Secondly, most reviews of screening tests for pre‐eclampsia and stillbirth do not account for the effect of intervention bias, because earlier scheduled birth and aspirin prophylaxis may prevent these outcomes. In such cases, conventional sensitivity/specificity analysis should be replaced with a competing risk approach to account for the effect of censorship and truncation of data. Finally, most public health specialists adhere to an effectiveness threshold for tests such as a likelihood ratio of >10 before recommending their use in clinical practice. Such arbitrary thresholds place perfection in the way of progress and constrain pregnancy risk assessment to a checklist‐based system that was developed over five decades ago. We have demonstrated that, in a large routine public healthcare setting, we may effectively improve pregnancy outcomes and tackle health inequality with modest organisational change and limited additional resources.^13^, ^14^

### 4.3 Strengths and limitations

This is the first study systematically to evaluate the impact of first trimester FMF combined screening for placental dysfunction on rates of perinatal death stratified by maternal ethnicity. To detect whether the intervention had a significantly greater effect than any temporal confounding factor due to an underlying secular trend, an interrupted time series analysis would have been optimal, but the number of index events precluded such an analysis. However, the study comparisons and significant findings for perinatal death within each cohort (by ethnicity, SGA and HDP) in the same time period, should be free of such secular trends. It is possible that other improvements in general clinical practice, which may have previously been worse for some minorities, may have contributed to the study effect. However, maternity care provision in the UK is not tailored to maternal ethnicity, except for the study intervention of first trimester screening for pre‐eclampsia, reducing the likelihood of this limitation. For example, even though we did not formally assess aspirin compliance, we have previously reported that aspirin prescription was only 29% in the NICE cohort compared with 99% for the FMF cohort.^13^, ^14^ This difference in care provision is directly related to first trimester assessment of pre‐eclampsia risk and not general care provision. Furthermore, about 40% of perinatal losses were excluded from the analysis because they were a consequence of fetal structural/genetic abnormality or due to *in utero* transfer just prior to birth—with outcomes unlikely to be modified by routine early pregnancy screening. To assess reliably the significance of the impact on perinatal death rates following implementation of the FMF combined screening programme, a larger multicentre collaborative study is required.

## 5 CONCLUSIONS

First trimester combined screening for placental dysfunction is associated with a significant reduction in perinatal deaths for women of Black, Asian and minority ethnic background and equitable health outcomes for non‐White and White women. An estimated 21 perinatal deaths were avoided in 4349 non‐White women during the study period. The likely mechanism of action is through the early and accurate assessment of the risk of placental dysfunction, resulting in women being allocated to the correct care pathway depending on their personalised risk assessment. Currently observed health inequality for perinatal death among ethnic minority women demands urgent attention from both clinicians and health policy makers. This study suggests that such health disparity is potentially avoidable.

### DISCLOSURE OF INTERESTS

Prof. Basky Thilaganathan sits on the Fetal Maternal and Child Health Subcommittee of the National Screening Committee of Public Health England.

## AUTHOR CONTRIBUTIONS

Conceptualisation: BT. Methodology: BL, BT. Data collection and processing: BL, UN. Statistical analysis: BL, UN, AF. Data interpretation: BL, AF, BT. Manuscript draft: BL, BT. Manuscript review and editing: BL, AF, MA, AB, BT.

## DETAILS OF ETHICS APPROVAL

This retrospective study of routinely collected clinical data was collated from ongoing continuous audit and was deemed not to require ethics approval or signed patient consent as per the HRA decision tool.

## Supporting information


Data S1

Data S2

Data S3

Data S4

Data S5

Data S6

Figure S1

Figure S2

Figure S3

Table S1

## Data Availability

The data that support the findings of this study are available on request from the corresponding author. The data are not publicly available due to privacy or ethical restrictions.
