# Supplementary material for: Reducing health inequality in Black, Asian and other minority ethnic pregnant women: impact of first trimester combined screening for placental dysfunction on perinatal mortality
Source: BJOG. 2022 Feb 27;129(10):1750–6. doi: 10.1111/1471-0528.17109 (PMC9544950; doi:10.1111/1471-0528.17109)
Supplement: Supplementary file 7 — Figure S1 [file BJO-129-1750-s010.docx]

All stillbirth and neonatal deaths

Gestation >24wks, Birthweight >300g

July 2016-Dec 2020

**n=179, PND rate=8.67/1000 births**

Excluding pregnancy terminations

**n=138**

**PND rate=6.68/1000 births**

Excluding known major congenital anomalies or genetic conditions

**n=102**

**PND rate=4.94/1000 births**

Excluding IUT occurring less than 4 weeks before birth

**n=71**

**PND rate=3.44/1000 births**

Stillbirth **n=55**

**SB rate=2.66/1000 births**

Neonatal deaths **n=16**

**NND rate=0.77/1000 births**

**Figure 1:** Flow chart showing numbers and rates (/1000 total births) of stillbirths (SB), neonatal (NND) and perinatal deaths (PND), before and after the exclusion of pregnancy terminations, major anomalies, and in-utero transfers (IUT) less than 4 weeks before birth that did not receive routine antenatal care at our unit.
