# Supplementary material for: Reducing health inequality in Black, Asian and other minority ethnic pregnant women: impact of first trimester combined screening for placental dysfunction on perinatal mortality
Source: BJOG. 2022 Feb 27;129(10):1750–6. doi: 10.1111/1471-0528.17109 (PMC9544950; doi:10.1111/1471-0528.17109)
Supplement: Supplementary file 8 — Figure S2 [file BJO-129-1750-s006.pptx]

## Slide 1
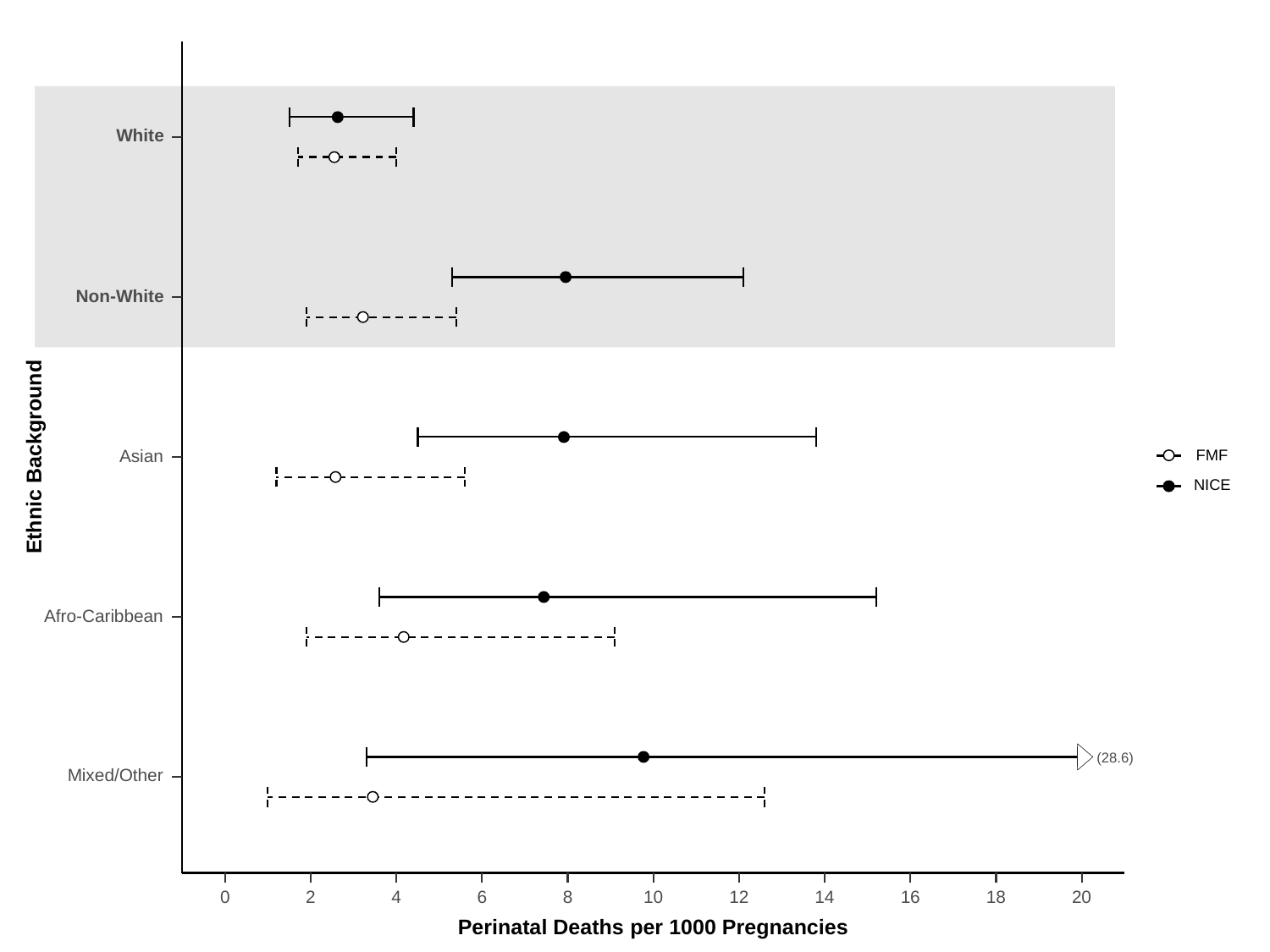

White
Non-White
Ethnic Background
FMF
Asian
NICE
Afro-Caribbean
(28.6)
Mixed/Other
8
18
20
6
16
0
10
2
12
4
14
Perinatal Deaths per 1000 Pregnancies
