# Supplementary figures and images for: Reducing health inequality in Black, Asian and other minority ethnic pregnant women: impact of first trimester combined screening for placental dysfunction on perinatal mortality
Source: BJOG. 2022 Feb 27;129(10):1750–6. doi: 10.1111/1471-0528.17109 (PMC9544950; doi:10.1111/1471-0528.17109)

## Slide 1
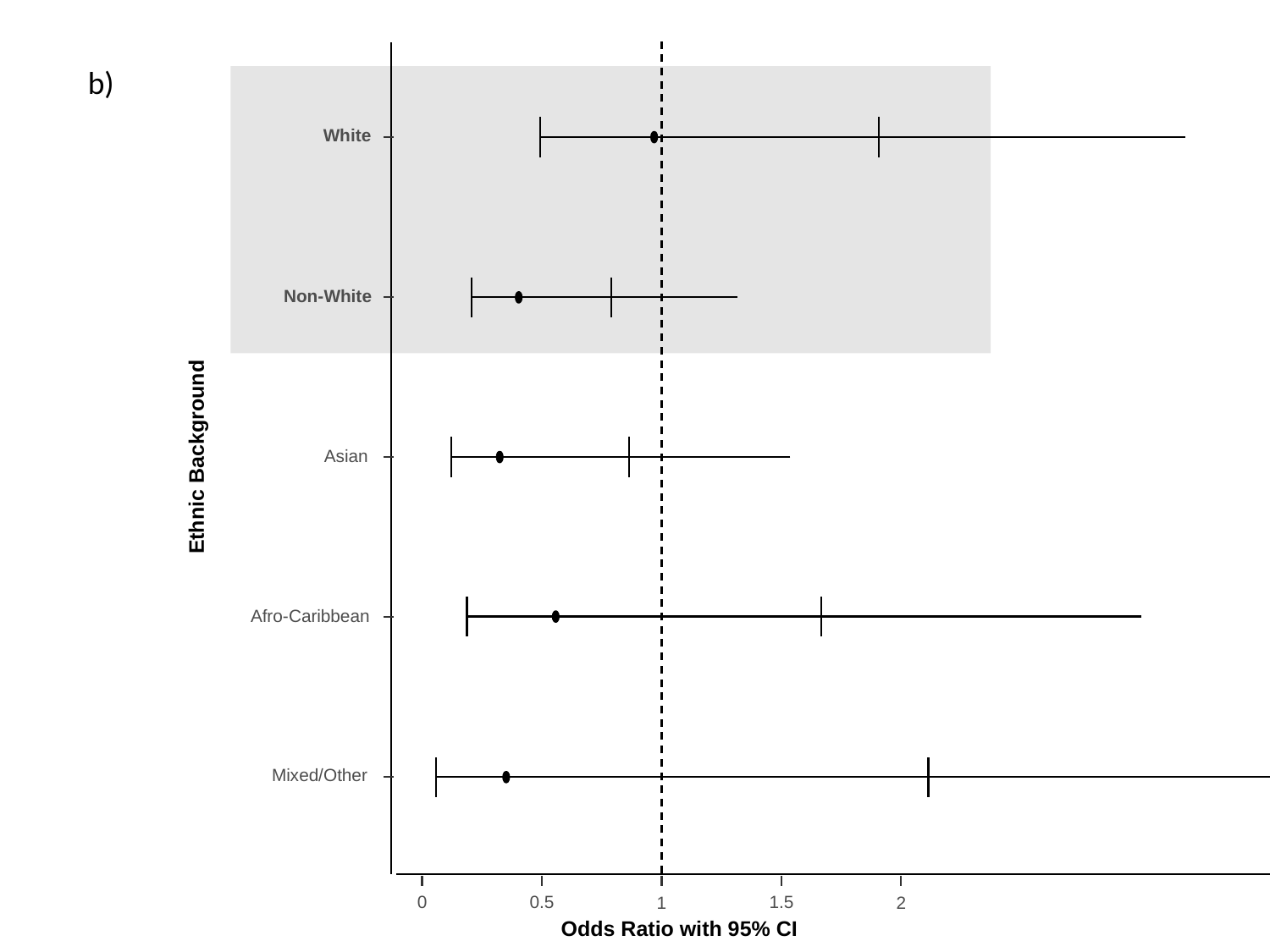

b)
White
Non-White
Asian
Ethnic Background
Afro-Caribbean
Mixed/Other
0
0.5
1.5
2
1
Odds Ratio with 95% CI

Supplement: Supplementary file 9 — Figure S3 [file BJO-129-1750-s002.pptx]
