# Supplementary material for: Reducing health inequality in Black, Asian and other minority ethnic pregnant women: impact of first trimester combined screening for placental dysfunction on perinatal mortality
Source: BJOG. 2022 Feb 27;129(10):1750–6. doi: 10.1111/1471-0528.17109 (PMC9544950; doi:10.1111/1471-0528.17109)
Supplement: Supplementary file 10 — Table S1 [file BJO-129-1750-s001.docx]

**Table S1:** Table comparing the number of perinatal deaths and perinatal death rates in women who underwent NICE and FMF screening, stratified by ethnicity. The odds ratios (9%% confidence intervals) are shown by type of screening method for each ethnic group.

|  | **NICE screened**  **(n=8080)** | **FMF screened**  **(n=12,571)** | **Odds ratio (95% CI)** |
| --- | --- | --- | --- |
| **White** | 14/5314  2.63 (1.5 to 4.4) | 21/8222  2.55 (1.7 to 4.0) | 0.969 (0.493-1.908) |
| **Non-white ethnicities** | 22/2766  7.95 (5.3 to 12.1) | 14/4349  3.22 (1.9 to 5.4) | **0.403 (0.206-0.789)** |
| Asian | 12/1519  7.91 (4.5 to 13.8) | 6/2330  2.58 (1.2 to 5.6) | **0.324 (0.121-0.865)** |
| Black | 7/941  7.44 (3.6 to 15.2) | 6/1440  4.17 (1.9 to 9.1) | 0.558 (0.187-1.666) |
| Mixed/other | 3/307  9.77 (3.3 to 28.4) | 2/579  3.45 (1.0 to 12.6) | 0.351 (0.058-2.114) |
